# Inflammatory Genital Infections Mitigate a Severe Genetic Bottleneck in Heterosexual Transmission of Subtype A and C HIV-1

**DOI:** 10.1371/journal.ppat.1000274

**Published:** 2009-01-23

**Authors:** Richard E. Haaland, Paulina A. Hawkins, Jesus Salazar-Gonzalez, Amber Johnson, Amanda Tichacek, Etienne Karita, Olivier Manigart, Joseph Mulenga, Brandon F. Keele, George M. Shaw, Beatrice H. Hahn, Susan A. Allen, Cynthia A. Derdeyn, Eric Hunter

**Affiliations:** 1 Department of Pathology and Laboratory Medicine, Emory University, Atlanta, Georgia, United States of America; 2 Emory Vaccine Center at Yerkes National Primate Research Center, Emory University, Atlanta, Georgia, United States of America; 3 Department of Medicine, University of Alabama at Birmingham, Birmingham, Alabama, United States of America; 4 Department of Global Health, Rollins School of Public Health, Emory University, Atlanta, Georgia, United States of America; 5 Projet San Francisco, Rwanda Zambia HIV Research Group, RZHRG, Kigali, Rwanda; 6 Zambia Emory HIV Research Project, ZEHRP, Lusaka, Zambia; 7 Zambia Blood Transfusion Service, Lusaka, Zambia; University Zurich, Switzerland

## Abstract

The HIV-1 epidemic in sub-Saharan Africa is driven largely by heterosexual transmission of non-subtype B viruses, of which subtypes C and A are predominant. Previous studies of subtype B and subtype C transmission pairs have suggested that a single variant from the chronically infected partner can establish infection in their newly infected partner. However, in subtype A infected individuals from a sex worker cohort and subtype B individuals from STD clinics, infection was frequently established by multiple variants. This study examined over 1750 single-genome amplified viral sequences derived from epidemiologically linked subtype C and subtype A transmission pairs very early after infection. In 90% (18/20) of the pairs, HIV-1 infection is initiated by a single viral variant that is derived from the quasispecies of the transmitting partner. In addition, the virus initiating infection in individuals who were infected by someone other than their spouse was characterized to determine if genital infections mitigated the severe genetic bottleneck observed in a majority of epidemiologically linked heterosexual HIV-1 transmission events. In nearly 50% (3/7) of individuals infected by someone other than their spouse, multiple genetic variants from a single individual established infection. A statistically significant association was observed between infection by multiple genetic variants and an inflammatory genital infection in the newly infected individual. Thus, in the vast majority of HIV-1 transmission events in cohabiting heterosexual couples, a single genetic variant establishes infection. Nevertheless, this severe genetic bottleneck can be mitigated by the presence of inflammatory genital infections in the at risk partner, suggesting that this restriction on genetic diversity is imposed in large part by the mucosal barrier.

## Author Summary

Previous studies of HIV transmission have yielded conflicting results regarding the genetic heterogeneity of the virus establishing infection in the newly infected individual. In this study of populations from Zambia and Rwanda that are infected by two distinct viral genetic subtypes, we compared viral sequences that encode the entry-mediating envelope glycoproteins from newly infected individuals (recipients) and their spouses (donors) very early after infection, as well as newly infected individuals infected by someone other than their spouse. In spite of the genetically diverse virus population in the donor, approximately 90% of newly infected individuals were infected by a single viral variant, while the rest were infected by multiple viral variants. The homogeneity of the virus population in the newly infected recipient, as well as the presence, in some cases, of identical virus variants in the donor, allowed us to precisely identify the transmitted variant. We were able to examine the clinical history of each newly infected individual and observed that all individuals infected by multiple variants also showed evidence of inflammatory genital infections. Our results suggest that the genital mucosa provides a natural barrier to infection by multiple genetic variants of HIV-1, but that this barrier can be lowered by inflammatory genital infections.

## Introduction

Nearly 35 million people across the globe are infected with the human immunodeficiency virus type 1 (HIV-1) and an additional 2.5 million new infections occur annually [1]. The current pandemic is the result of viruses that are genetically diverse and have been divided into 9 different subtypes (A–D, F–H, J, K) and at least 28 circulating recombinant forms (www.hiv.lanl.gov). Subtype B viruses predominate in North America and Western Europe, and as a result, research efforts have focused primarily on this subtype. However, almost 25 million individuals in sub-Saharan Africa have been infected with non-subtype B viruses via heterosexual transmission, leading to a significant gap in our understanding of the most predominant HIV-1 variants worldwide. In order to achieve protection against infection with a globally effective vaccine, the origin and nature of variants that establish a new infection must be better defined.

The mechanisms by which HIV-1 is transmitted sexually across a mucosal barrier remain poorly understood. Characterization of viral sequences in newly infected individuals has produced mixed results. For instance, studies of individuals newly infected with subtype B have demonstrated a relatively homogenous viral population, suggesting infection by a single variant [2],[3],[4]. In contrast, studies involving subtype-B virus transmission in a population harboring sexually-transmitted diseases and subtype-A virus in a sex-worker cohort suggest multiple viral variants can be transmitted and are capable of establishing infection in a new host [5],[6],[7],[8]. Thus, the viral correlates of transmission are likely to be influenced by subtype, study population, and route of infection. Since only the newly transmitted virus population was characterized in the majority of these studies, no information was available regarding its relationship to the virus circulating in the transmitting partner. Nevertheless, these studies indicate that either a single viral variant or multiple variants can be transmitted and establish a new HIV infection.

A previous study in this laboratory examined 8 epidemiologically linked heterosexual transmission pairs participating in a HIV-discordant couple cohort in Lusaka, Zambia [9]. Seven of these pairs harbored subtype C viruses, while the remaining pair was infected with subtype G. In each transmission pair, comparison of the virus population derived from the chronically infected (donor) and newly infected partners (recipient) revealed a severe genetic bottleneck during heterosexual transmission of HIV, which was characterized by low sequence diversity in the recipient. Moreover, the monophyletic nature of each recipient virus population relative to the donor quasispecies indicated that each was derived from a single variant within the donor.

Here we have expanded our studies of HIV-1 transmission to include 12 additional transmission pairs from the Lusaka cohort and 8 pairs from a similar cohort in Kigali, Rwanda [10],[11]. The predominant circulating subtypes in Lusaka and Kigali are subtype C and subtype A respectively, providing a unique opportunity in which to investigate the correlates of heterosexual transmission in regions where the two most predominant HIV-1 subtypes circulate [12]. Each cohort is comprised of over 1,000 cohabitating HIV-discordant couples enrolled in a prospective prevention study in which participants return at 3-month intervals for preventive counseling and condom provision. Despite these interventions, a low frequency of HIV-1 transmission still occurs [13]. In the present study, plasma samples from HIV seronegative partners were tested at each visit for the appearance of antibodies to HIV-1 as well as for the presence of p24 antigen to identify acutely infected individuals in whom virus is present in the peripheral blood but antibody levels are still undetectable [14],[15],[16]. Moreover, the studies presented here utilized end-point dilution PCR (or single genome amplification (SGA)) to compare partial *env* gene sequences within the quasispecies of the donor and recipient. To investigate transmission in a setting analogous to those that are associated with transmission of multiple variants, we included a limited number of epidemiologically unlinked transmission events, in which the seronegative partner was infected by someone other than their spouse. The results indicate that, in the majority of cases, HIV-1 infection is initiated by a single viral variant from a complex quasispecies in the transmitting partner. The marked reduction in genetic diversity that is observed during HIV-1 transmission appears to be imposed in large part by the mucosal barrier, since this extreme genetic bottleneck can be mitigated when inflammatory infections are present in the recipient partner.

## Results

### A Severe Genetic Bottleneck Occurs During Subtype A and C HIV-1 Transmission

HIV envelope sequences from twenty epidemiologically linked heterosexual transmission pairs from the Lusaka and Kigali cohorts were used for comparative sequence analysis of the donor and recipient viruses (Table 1). Consistent with previous studies [9],[17], 10/12 transmission pairs from the Lusaka cohort were identified as harboring subtype C envelope sequences. The remaining two pairs were infected with subtype A (ZM292) and a virus that could not be classified (ZM248), since *env* sequences from the latter formed a distinct cluster that is equidistant from known subtypes (data not shown). All of the 8 transmission pairs from the Kigali cohort were identified as encoding subtype A Env sequences (data not shown). Importantly, 14/20 of the newly infected partners were identified as p24-positive or viral RNA-positive prior to or on the date of seropositive testing and sample collection from both partners, and for 11 of these subjects, the samples analyzed were collected within an estimated 3–5 weeks (17–40 days) following infection (Fiebig stages II–IV, [18]). These pairs thus represent very recent transmission events (Table 1).

**Table 1:** Samples Collected for Analysis.

Recipient ID	Sample Date	Estimated Days from Infection	MRCA (Days)	Envelope Subtype
**Linked to Enrolled Partner**				
ZM229M	19-Oct-02	<94	1409	C
ZM238M*	29-Oct-02	40	5	C
ZM242F*	25-Jan-03	31	30	C
ZM201M*	7-Feb-03	31	35	C
ZM221M*	7-Mar-03	31	23	C
ZM205F*	27-Mar-03	48	19	C
ZM248F*	5-Jun-03	79	108	Unknown
ZM190F*	6-Dec-03	31	15–30+	C
ZM216M*	17-Jan-04	31	66	C
ZM198F*	4-Mar-04	78	126	C
ZM243F*	9-Mar-04	26	24	C
RW41M	21-Jan-05	<94	26–52+	A1
RW19F	2-Feb-05	<96	26–35+	A1
RW35M	28-Feb-05	<96	47–59+	A1
RW36M	7-Mar-05	<86	21	A1
RW56F#	21-Apr-05	17	18	A1
ZM292M*	24-May-05	28	44	A1
RW53F*	29-Jun-05	35	35	A1
RW57F	12-Oct-05	<94	166	A1
RW67M*	23-Mar-06	37	41	A1
**Unlinked to Enrolled Partner**				
ZM215F	3-Oct-02	<92	2625	C
ZM197M#	29-Oct-02	17	17	C
ZM224F	5-Nov-02	<134	568	C
ZM233M	17-Dec-02	<110	36	C
ZM184F	10-Jul-03	<92	31	C
ZM249M*	12-Aug-03	29	21	C
ZM247F*	1-Nov-03	26	536	C

*: Individuals identified as p24-positive.

# Individuals identified as viral RNA-positive.

**+:** Range of values indicate a higher than predicted rate of G to A mutations consistent with APOBEC3G/F signatures.

**Figure 1:**
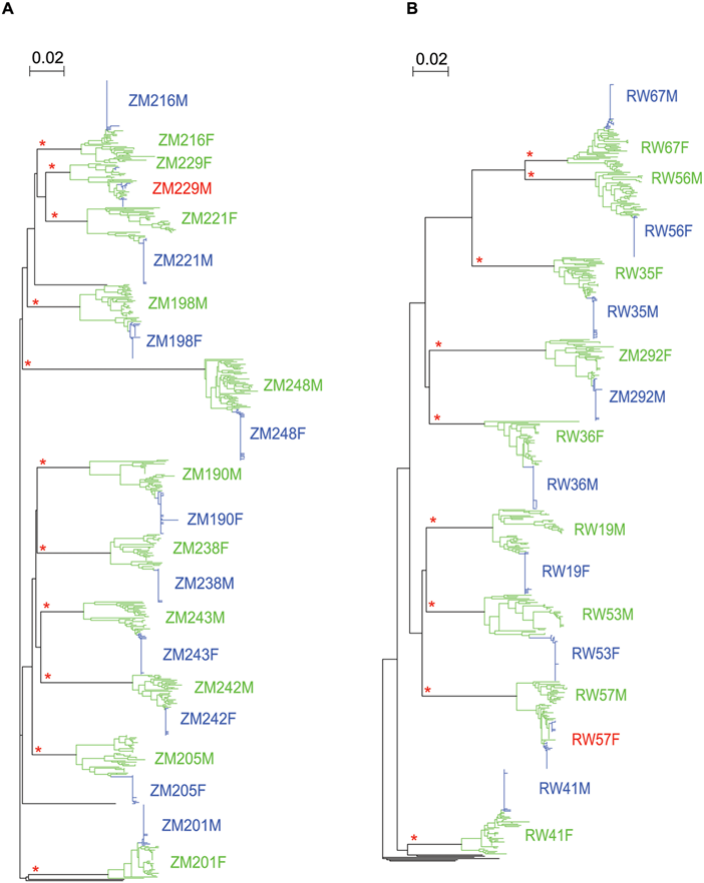
Phylogenetic analysis of heterosexual transmission pairs. Nucleotide sequences were aligned for all linked donors (green lines) and linked recipients (blue lines) and neighbor-joining trees were drawn representing (A) subtype C transmission pairs and (B) subtype A transmission pairs. Recipients in red indicate infection by multiple variants. Horizontal branch lengths are drawn to scale with scale bar representing 2% divergence. Asterisks indicate branches with bootstrap values greater than 0.99. Black lines represent unrelated reference sequences.

**Figure 2:**
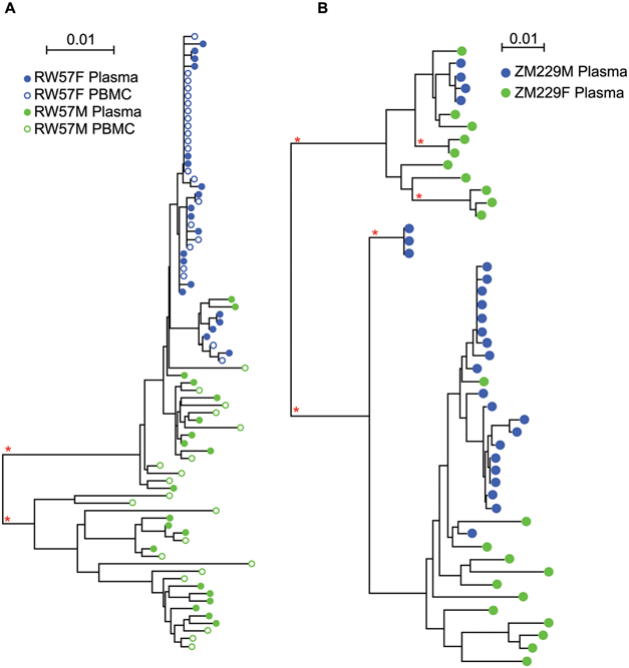
Transmission of multiple variants. Aligned nucleotide sequences for linked donors (green circles) and linked recipients (blue circles) were used to generate neighbor-joining trees for individual transmission pairs (A) RW57 and (B) ZM229. Horizontal branch lengths are drawn to scale with scale bar representing 1% divergence. Open circles represent sequences derived from uncultured PBMC DNA and closed circles represent sequences derived from plasma RNA. PBMC samples were unavailable for ZM229; therefore, only plasma sequences were derived for this transmission pair. Asterisks indicate branches with bootstrap values greater than 0.99.

For each partner of a transmission pair, approximately 40 HIV-1 *env* genes were amplified from uncultured PBMC DNA (20 amplicons) and plasma virus RNA (20 amplicons). SGA was employed to minimize the potential for *in vitro* recombination during the PCR reaction and re-sampling of genomes [19],[20]. Each amplicon was directly sequenced over the V1–V4 region of the HIV-1 envelope, the most variable region of the *env* gene. Neighbor-joining phylogenetic trees were constructed using the sequences derived from the 10 subtype C transmission pairs and ZM248 (subtype unknown) (Figure 1A) and the 9 subtype A transmission pairs (Figure 1B). Sequences from each donor-recipient pair clustered together with high bootstrap support, indicating a lack of cross-contamination from other samples or the presence of related transmission networks. In each case the donor sequences were heterogeneous, consistent with their derivation from a chronically infected individual. Sequences from PBMC DNA and plasma virus RNA were distributed throughout the branch patterns of each donor and recipient, suggesting a lack of observable compartmentalization between these two sources (Figure 2A). In 18 of the 20 transmission pairs, recipient envelope sequences were homogenous forming a distinct monophyletic subcluster within the branch pattern of the donor sequences. This is consistent with our previous study [9] demonstrating that a single genetic variant from the donor quasispecies initiates infection in the recipient and with more recent analyses of subtype B HIV-1 acutely infected individuals [21].

In contrast, recipient sequences in the remaining two pairs (RW57 and ZM229) were not monophyletic. Examination of the neighbor-joining tree for RW57 (Figure 2A) reveals 2 distinct, but highly related subclusters of viral sequences for RW57F that are derived from a subset of RW57M sequences. The two variant populations in RW57F differ by a cluster of five synonymous mutations in the V1 region of gp120 that differentiate the seven sequences of cluster 2 from the 35 in cluster 1 (Figure S1A). Moreover, these signature sequences in cluster 2 are shared by two of the donor *env* genes, indicating that they did not arise de novo in the recipient. The branching pattern and the presence of two distinct populations of sequences that cannot be explained by viral evolution (see below), suggests that 2 different but closely related viral variants were involved in the establishment of infection in RW57F. The neighbor-joining tree for couple ZM229 (Figure 2B) showed recipient sequences forming 4 distinct branches within the donor sequences. This branching pattern again suggests that multiple viral variants, 4 in this case, were involved in establishing the infection in ZM229M. This is consistent with the analysis of the recipient sequences reported by Salazar et al. [20]. Thus, while a single donor variant establishes infection in 90% of the transmission events, RW57F and ZM229M demonstrate that multiple variants can be transmitted and establish infection. Moreover, these two cases demonstrate that a single most-fit genetic variant does not necessarily evolve to dominate the acute viral population.

**Figure 3:**
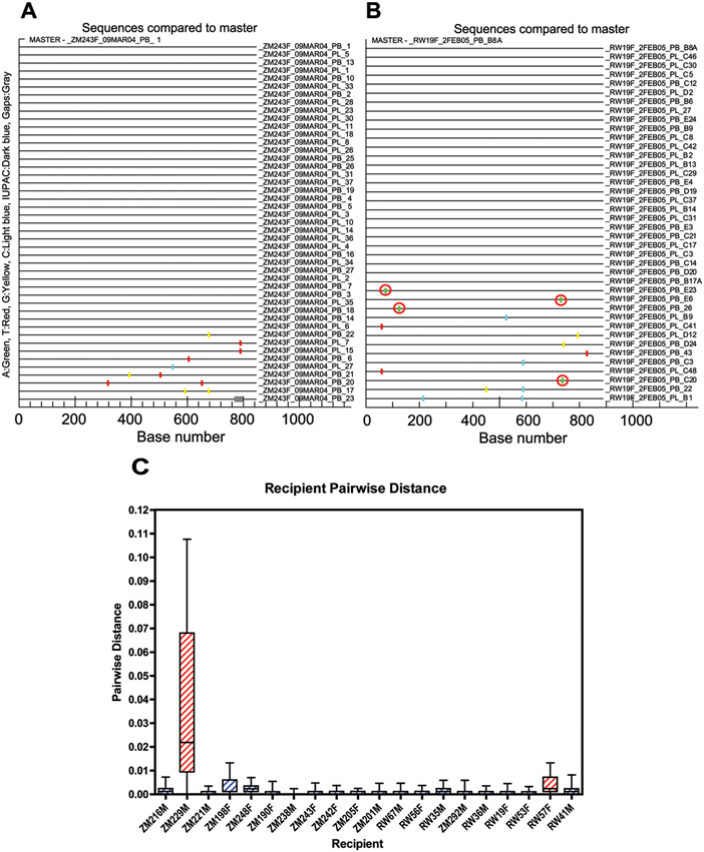
Homogenous virus population in newly infected individuals. Aligned linked recipient sequences were analyzed by the Highlighter tool (Los Alamos National Laboratory website - HIV Sequence Database), examples of output files are shown for (A) ZM243F and (B) RW19F. Tic marks indicate nucleotide differences from the indicated master sequences derived from the recipient. Nucleotide differences are color-coded and are marked according to their genetic location along the length of V1–V4. Colors are as follows: A: green, T: red, G: yellow, C: blue and gaps: gray. Tics highlighted by circles represent G to A changes in a sequence consistent with an APOBEC3G/F signature. (C) Box plots were generated using the pairwise distances calculated for individual linked recipients. Horizontal lines within box plots indicate median pairwise distance values for each linked recipient. Red boxes indicate individuals infected by multiple genetic variants.

To evaluate the extent of sequence diversity in the newly infected individuals, V1–V4 sequences from the recipient were examined using the Highlighter tool on the Los Alamos National Laboratory website. This tool allows a comparison of each recipient Env sequence to a reference recipient sequence and graphically depicts any nucleotide differences between the two. Examples of the output from this tool for one subtype C and one subtype A recipient are given in Figure 3A and 3B. A remarkable degree of homogeneity is observed in the V1–V4 sequences for ZM243F (Figure 3A), where infection was estimated to have occurred 26 days prior to sample collection and where approximately 80% (36 of 45) of the sequences are identical despite each being amplified from a unique viral genome. Similar numbers of identical sequences were observed in both plasma (21) and PBMC (15) derived sequences. Compared to the reference amplicon (PB_1), approximately 11% of the sequences exhibited a single nucleotide change randomly dispersed over the V1–V4 region, and 7% two nucleotide changes. One amplicon (PB_23) exhibited a three-codon deletion in the V4 region. Similarly, for RW19F (Figure 3B), where infection was estimated to be 26–35 days before sample collection (see below), approximately 68% of the sequences are identical, 28% contained a single base change and 5% two nucleotide changes compared to the reference amplicon (PB_B8A). These frequencies are consistent with a model where infection is initiated by a single donor genetic variant that undergoes base mis-incorporation at a rate of 1.7×10^−5^ per day or 3.4×10^−5^ per replication cycle [20]. The model used to calculate the most recent common ancestor is based on this observation and yields a Poisson distribution of mutations with star-like phylogeny [21].

For each set of the recipient sequences, we calculated the number of days required to explain the observed within patient *env* diversification from a single most recent common ancestor (MRCA) sequence as described previously (Table 1, [21]). Four of the recipients (ZM190F, RW19F, RW35M, RW41M) showed a higher than predicted rate of G to A mutations that occurred in sequences consistent with APOBEC3G/F signatures. For these individuals we calculated the time to MRCA with and without these mutations and expressed the time frame as a range of days. This is exemplified by Figure 3B where 4 G to A mutations (circled) occurred in sequences consistent with an APOBEC signature. A majority (16/20) of the calculated days from the MRCA agreed closely with the estimated time from infection based on patient history and Fiebig stage of infection [18]. By contrast, both ZM229 and RW57 showed MRCA calculations that exceeded the estimated time to infection consistent with the transmission of multiple variants as identified through phylogenetic and Highlighter analyses (Figure 2, Figure S1A and B). This was particularly notable for ZM229, where the MRCA calculation, which exceeds 1400 days, reflects the genetic heterogeneity of infection by multiple variants. Two other individuals, ZM216 and ZM292, exhibited times to MRCA double that estimated from their Fiebig stage of infection. Approximately one third of the Env sequences from patient ZM216 showed evidence of early CTL escape, characterized by a cluster of non-synonymous mutations in a single 9 amino acid region (Figure S2A), similar to those we have described previously [20] – similar CTL-escape footprints were observed in a smaller fraction of sequences in ZM248F, RW41M, and RW35M. For ZM292M approximately one third of the *env* sequences exhibited a common 2-codon deletion in the ß14 region of gp120 (Figure S2B), raising the possibility that two closely related variants were associated with transmission. However, based on the fact that all of the ZM292M sequences are derived from a single branch of the donor phylogenetic tree and that this deletion was not present in any of the 40 donor sequences, we hypothesize that this variant represents an early stochastic RT error that conferred some fitness advantage during acute infection.

To quantify the genetic variation observed for the viral quasispecies within each of the newly infected recipients, the pairwise distance was calculated for the nucleotide sequences in each recipient virus population (Figure 3C). As might be predicted from the phylogenetic analyses, in 19 of the 20 recipient virus populations the calculated median pairwise distance was extremely low, and in all but the 4 cases where CTL escape or APOBEC signatures were evident even the most divergent variants in the population differ by less than 1%. In the case of RW57F, the more divergent variants were consistent with more than one closely related variant being transmitted. The most dramatically different pattern observed was with ZM229M, for which the median pairwise distance was over 2%, and the most divergent variants differed by almost 11%, consistent with multiple distinct variants from a single donor being involved in establishing infection.

Taken together, these results suggest that in a majority of cases where heterosexual transmission occurs within an HIV-1 discordant couple, a severe genetic bottleneck restricts viral diversity, with a highly homogeneous population representative of the initiating virus variant predominating early after infection.

### Relationship of Recipient Viruses to the Donor Quasispecies

Because in a majority of cases a single donor variant establishes infection, we sought to determine the frequency of this variant or closely related variants in the donor quasispecies and to examine whether such variants predominated in plasma or PBMC derived viruses in the donor. The application of single genome PCR amplification and analysis of approximately 20 plasma and 20 PBMC viruses for each donor allows this question to be approached with increased confidence. The number of amino acid differences between each donor virus sequence and the consensus recipient virus sequence were calculated for each of the 10 linked transmission pairs where Fiebig stage and MRCA calculations estimated infection to have occurred less than 50 days previously. In 8 of the 10 transmission pairs analyzed, at least one donor variant was identified that differed by fewer than 5 amino acids from the consensus recipient variant over the approximately 250 amino acid V1–V4 region (Table 2). In two cases, ZM221M and RW53F, a single viral variant in the donor quasispecies was identified whose amino acid sequence is identical to the consensus observed in the recipient, and in one other case (ZM242F) the most closely related donor variant differed by 1 amino acid from the recipient consensus. For these ten transmission pairs, the donor variants most closely related to the recipient sequences were derived from both plasma (4/10) and PBMC (6/10) samples. Therefore, in terms of the peripheral blood, it is not possible to establish whether the infecting virus is specifically derived from a single compartment – for example latently infected PBMC.

**Table 2:** Relationship of donor variants to recipient consensus sequence.

Recipient ID	Number of Donor Variants Analyzed	Number of Amino Acid Differences from Recipient	Blood Compartment of Origin of Nearest Donor Variant	Number of Donor Sequences <5 amino acids different
ZM221M	36	0	PBMC	1
RW53F	39	0	PBMC	1
ZM242F	37	1	Plasma	6
ZM238M	38	2	PBMC	1
ZM243F	36	3	Plasma	2
RW56F	40	3	PBMC	1
RW67M	41	3	Plasma	2
ZM201M	37	4	Plasma	1
ZM190F	35	10	PBMC	0
ZM205F	45	11	PBMC	0

The number of variants identified within each donor that contain fewer than 5 amino acid differences was determined for each transmission pair (Table 2). In 9 of the 10 transmission pairs, 2 or fewer variants (less than 5% of the donor sequences) were closely related (>98%) to variants identified in the recipient. This analysis suggests that in most transmission pairs, the virus establishing infection is derived from an infrequent variant population within the donor sequences derived from blood at the time samples were collected.

### Mitigation of the Severe Genetic Bottleneck

**Figure 4:**
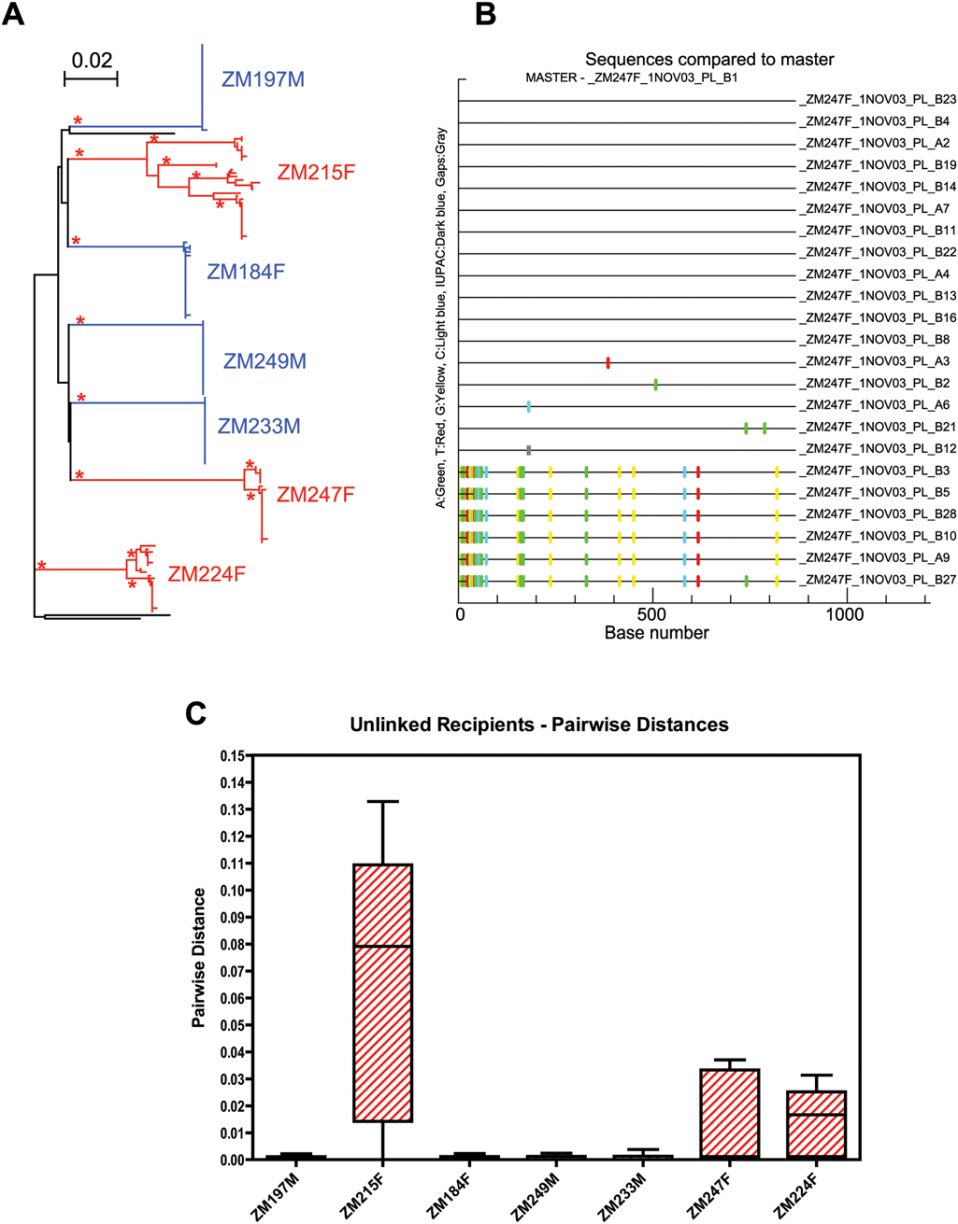
Analysis of unlinked recipients. (A) Aligned nucleotide sequences for 8 unlinked recipients were used to generate a neighbor-joining phylogenetic tree. Horizontal branch lengths are drawn to scale, with scale bar representing 2% divergence. Lines drawn in blue indicate individuals infected by a single variant, those in red indicate infection by multiple variants. Asterisks indicate branches with bootstrap values greater than 0.95. Black lines indicate unrelated reference sequences. (B) Aligned linked recipient sequences were analyzed by the Highlighter tool (Los Alamos National Laboratory website - HIV Sequence Database), an example of the output file is shown for ZM247F. Tic marks indicate nucleotide differences from the indicated master sequences derived from the recipient. Nucleotide differences are color-coded and are marked according to their genetic location along the length of V1–V4. Colors are as follows: A: green, T: red, G: yellow, C: blue and gaps: gray. (C) Box plots were generated using the pairwise distances calculated for individual unlinked recipients. Horizontal lines within box plots indicate median pairwise distance values for each unlinked recipient. Red boxes indicate individuals infected by multiple genetic variants.

In contrast to the results presented above, analyses of newly infected sex workers in Kenya and STD clinic participants in North Carolina suggested that, in up to 50% of cases, multiple variants established new infections [5],[6],[7],[8]. This raised the question of whether the factors involved in epidemiologically linked transmissions in the discordant couple cohorts are intrinsically different from more broadly exposed populations. Because in the Kenya studies a link existed between sexually transmitted infections (STIs) and virus heterogeneity in the newly infected participant, we analyzed a subset of newly infected individuals within the cohort who had been infected outside the cohabiting partnership, and who might therefore be at higher risk of STIs. A total of 7 epidemiologically unlinked recipients were identified from the Lusaka cohort for sequence analysis of the HIV envelope (Table 1). Three of the 7 unlinked individuals were identified as p24-positive or virus RNA positive and samples were obtained very near the time of infection. Single genome amplification was used to generate approximately 20 HIV-1 envelope amplicons from plasma virus RNA of each unlinked recipient. Each envelope amplicon was directly sequenced over the V1–V4 region and a neighbor-joining phylogenetic tree was constructed from the sequences (Figure 4A). Viruses in each of the 7 unlinked recipients were identified as subtype C (data not shown) and their *env* sequences were monophyletic indicating that each had been derived from a single unidentified donor partner. The sequences in four of the unlinked recipients were highly homogenous (ZM184F, ZM249M, ZM197M, ZM233M) similar to that observed in the linked recipients. In contrast, three of the unlinked recipients (ZM224F, ZM247F, ZM215F) have a highly diverse virus population, where multiple divergent branches appear to be present. The Highlighter Tool output for ZM247F is shown in Figure 4B and clearly displays two distinct variants that exhibit a large number of nucleotide differences from each other within the newly infected individual, consistent with at least 2 viral variants being involved in the establishment of infection in this individual. Similar analyses for ZM224 and ZM215 (Figure S1C and D) indicate that in each of these cases at least two distinct variants from a single unidentified donor initiated infection in the unlinked recipient.

To further quantitate the genetic diversity in the seven unlinked individuals, nucleotide pairwise distances were calculated for the sequences within each subject (Figure 4C). As might be predicted from the phylogenetic analysis, the pairwise distance calculations for ZM224F, ZM247F and ZM215F show that the virus quasispecies in each of these recipients are very heterogeneous, differing from each other by as much as 13%. This is a level of heterogeneity that far exceeds that predicted from RT-based misincorporation within the maximum estimated time from infection. The 4 remaining unlinked recipients harbored highly homogeneous virus populations with less than 0.5% divergence. We also performed MRCA calculations for the seven unlinked individuals as was performed for the linked recipients (Table 1). The four unlinked individuals with highly homogeneous virus (ZM184F, ZM249M, ZM197M, ZM233M) showed MRCA calculations consistent with the estimated time of infection. However, ZM224F, ZM247F and ZM215F exhibited time-to-MRCA calculations that were between 4 and 28 times longer than the estimated time from infection (Table 1). This is again consistent with the level of heterogeneity observed in the phylogenetic, Highlighter and pairwise-distance analyses. Taken together these results suggest that in 3 of the 7 unlinked recipients (42.9%), infection was established by multiple viral variants. While the numbers of individuals analyzed is small, this does represent a frequency significantly higher than that observed in the linked transmission pairs (Table 3; p = 0.0211). Initial studies in a Kenyan sex worker cohort suggested that heterogeneous virus populations were more common in newly infected females [5], but additional studies showed evidence of heterogeneous virus populations in both newly infected females and males [8]. An analysis of the association of multiple variants and gender in this study did not reveal any statistical significance (Table 3).

**Table 3:** Multiple Variants Association.

	Single Variant		Multiple Variants		p value*
	n	%	n	%	
**Gender of seroconverter - Lusaka**					
Female	17	54.8%	3	75.0%	0.6272
Male	13	41.9%	1	25.0%	
**Gender of seroconverter - Kigali**					
Female	3	42.9%	1	100.0%	1.0000
Male	4	57.1%	0	0.0%	
**Linkage - Lusaka**					
Unlinked	4	12.9%	3	75.0%	***0.0211***
Linked	26	83.9%	1	25.0%	

*: p-value calculated using Fisher's exact test.

Thus, in the context of both linked and unlinked transmissions, conditions do exist that mitigate the genetic bottleneck, allowing for transmission of multiple variants. However, this occurred more frequently in the unlinked recipients. An analysis of the clinical history for each of the 7 unlinked recipients showed a significant association (p = 0.0286) between the presence of vaginal/urethral discharge, which is evidence of an inflammatory genital infection, and the establishment of infection by more than one genetic variant in the three females in which this was present (Table 4). Lower abdominal pain in the newly infected recipient was also significantly associated with multiple genetic variants establishing infection in the unlinked recipients (p = 0.0286; Table 4).

**Table 4:** Prospective evaluation of clinical signs/symptoms of STIs on the day of and last seronegative visit prior to seroconversion.

	Zambia Unlinked					Zambia+Rwanda				
	Single (N = 4)	%	Multiple (N = 3)	%	p-value*	Single (N = 37)	%	Multiple (N = 5)	%	p-value*
**Genital inflammation**										
No	3	75.0%	0	0.0%	0.1429	25	67.6%	2	40.0%	0.3295
Yes	1	25.0%	3	100.0%		12	32.4%	3	60.0%	
**Genital ulceration**										
No	3	75.0%	2	66.7%	1.0000	29	78.4%	2	40.0%	0.1028
Yes	1	25.0%	1	33.3%		8	21.6%	3	60.0%	
**Genital inflammation or ulceration**										
No	2	50.0%	0	0.0%	0.4286	20	54.1%	0	0.0%	***0.0492***
Yes	2	50.0%	3	100.0%		17	45.9%	5	100.0%	
**Discharge**										
No	4	100.0%	0	0.0%	***0.0286***	28	75.7%	2	40.0%	0.3249
Yes	0	0.0%	3	100.0%		9	24.3%	3	60.0%	
**Cystitis** a										
No	4	100.0%	2	66.7%	0.4286	31	88.6%	4	80.0%	0.5066
Yes	0	0.0%	1	33.3%		4	11.4%	1	20.0%	
**Lower abdominal pain (self report)** a										
No	4	100.0%	0	0.0%	***0.0286***	31	88.6%	2	40.0%	***0.0299***
Yes	0	0.0%	3	100.0%		4	11.4%	3	60.0%	

*: p-value calculated using Fisher's exact test.

a 2 Zambia subjects missing data on cystitis and lower abdominal pain because seroconversion detected between screening and enrollment visits.

This analysis of clinical histories was expanded to include a total of 42 newly infected individuals for whom we had established whether infection in the recipient was initiated by a single or multiple genetic variants from the donor, in order to identify any significant associations between clinical criteria and the establishment of infection by more than one genetic variant. This group included the 27 newly infected individuals presented here, and fifteen, additional linked recipients that have been described [9],[21]. Interestingly, when these 42 recipients were subjected to analysis, all 5 recipients characterized as having been infected by multiple genetic variants showed evidence of genital inflammation (3 females) or ulceration (1 male, 2 female) (Table 4). Moreover, the presence of genital inflammation or ulceration was significantly associated with the presence of multiple genetic variants in the recipient (p = 0.0492). Similarly, the presence of lower abdominal pain in the recipient was also significantly associated with multiple variants establishing infection (p = 0.0299) (Table 4).

## Discussion

### A Severe Genetic Bottleneck During Heterosexual Transmission of HIV-1

In studies that have characterized the virus population in newly infected individuals, the complexity of the virus is higher in some cohorts than in others. Most of these studies were unable to examine the viral population of the sexual partner of that individual. The samples used in the studies presented here were collected from both the chronically infected donor and the newly infected recipient partner enrolled in discordant couple cohorts in Lusaka, Zambia and Kigali, Rwanda. This has provided a unique opportunity to compare epidemiologically linked virus populations in both the donor and recipient very near the time of virus transmission and to use this information to define the nature and origin of the virus establishing infection. The studies presented here reinforce our previous finding that a severe genetic bottleneck occurs during heterosexual transmission of subtype C HIV-1 [9]. In a majority (90%) of linked subtype C and subtype A transmission pairs, a single viral variant or a single virus infected cell established each new infection (Figure 1), as supported by phylogenetic and Highlighter analyses of donor and recipient viral sequences, as well as MRCA calculations for each recipient virus population. Thus, viruses circulating in 2 distinct geographic locations and ethnic communities exhibit a severe genetic bottleneck during heterosexual transmission, irrespective of virus subtype or host genetic background. Furthermore, our ability to examine the sequences from 10 individuals during acute infection demonstrated how remarkably homogeneous the virus population is at this time (Figure 3). The consensus sequence observed soon after infection can therefore inform us as to the variant that established infection in the new host. This is consistent with and supports the recent results of Keele et al. who have characterized early isolates of subtype B HIV-1 [21].

Variants could be identified within the donor quasispecies that were identical (ZM221 and RW53) or highly related (less than 2% amino acid differences) to the variant establishing infection in the recipient in several transmission pairs (Table 2). However, the “transmitted” donor variants generally represented a small fraction (less than 5%) of the donor quasispecies rather than being the predominant population circulating within the peripheral blood. An analysis of genetic variants in the donor's genital fluids in a subset of these same transmission pairs at the time infection was detected, has indicated that the virus that establishes infection, is in most cases, more related to blood-derived variants than those enriched in the genital compartment (Boeras et al., manuscript in preparation). In the present study, these “transmitted” donor variants were detected with approximately equal frequency among sequences from PBMC DNA or plasma viral RNA. Thus, within the sampling constraints of this large cohort study, we were unable to distinguish between the cell-free, plasma-derived virions and cell-associated virus as the source of the new infection. Nevertheless, the finding of identical and highly related variants within the donor confirms that the newly transmitted virus existed in the donor quasispecies prior to transmission, and therefore did not result from very early *de novo* viral evolution in the new host.

A previous study in this laboratory reported that the genetic bottleneck involved a selection for variants with length-constrained hyper-variable domains compared to the median of corresponding donor sequences [9]. In that study, viral sequences were predominantly derived from PBMC DNA to capture the entire spectrum of genetic diversity in the donor. An analysis of the single genome amplified viral sequences derived from PBMC DNA from the 10 subtype C transmission pairs presented here have yielded results consistent with the previous study in that the majority of recipient viruses encode more compact V1–V4 regions compared to the donor median (Haaland et al, manuscript in preparation). In contrast, a similar comparison of donor and recipient PBMC-derived Env sequences from the 9 subtype A transmission pairs described here and an additional 12 pairs analyzed independently showed no significant differences in V1–V4 (or V1–V2) length between donors and recipients (Haaland et al, manuscript in preparation). The subtype A results differ from a previous study of newly infected Kenyan sex workers, in which a statistically significant difference was observed in V1–V2 length compared to sequences from chronically infected individuals found in the Los Alamos National Laboratory's HIV Sequence Database [22]. These conflicting results raise the possibility that subtype A sequences from different geographical regions, such as Kenya and Rwanda, may have inherent differences in this regard.

### Mitigation of the Severe Genetic Bottleneck

In only a minority (10%) of the linked transmission events examined, were recipient sequences present on multiple branches of the donor phylogenetic tree (ZM229 and RW57; Figure 2), indicative of more than one donor variant being transmitted. In contrast, an analysis of 7 “unlinked” recipients from the Lusaka cohort suggested that transmission of multiple variants is more common in this setting (Figure 4). Three individuals were infected with at least two variants, and this represented a statistically significant increase in the frequency of infection by multiple variants in this study population compared to the linked transmission pairs (Table 3).

The criteria used to confirm that multiple genetic variants established infection are defined in detail in Protocol S1. These included (i) the absence of a Poisson distribution of mutations with star-like phylogeny resulting in MRCAs that significantly exceeded the maximum estimated date of infection, (ii) distinct populations of sequences in the highlighter analysis that could not be readily explained by selection of CTL escape variants, and (iii) in the case of transmission pairs more than one branch of recipient sequences emanating from the donor tree. While 4/5 of the individuals infected with multiple variants have an estimated date of infection that exceeds 90 days (Table 1), this reflected the lack of a p24 positive date with which to define a more accurate estimate of the time of infection and required that we instead calculate the time from the last seronegative visit to the first seropositive visit. Nevertheless infection could have occurred at any point during this period. Indeed for six individuals infected with a single variant for whom a p24 positive date was not available, and whose estimated date of infection exceeded 85 days, the MRCA estimates were between one third and one half the estimated time of infection (31–47 days; Table 1). Moreover, even the longest estimated time of infection could not explain the level of heterogeneity observed in the *env* sequences from these individuals or the presence of between two and five subsets of sequences in which the members exhibit multiple identical nucleotide differences (Figure 4 and Figure S1).

Since the exact time of infection is not known for any of the partners in the ZEHRP or PSF cohorts, we cannot definitively rule out the possibility that some of those individuals who were classified as infected by “multiple” variants may have in fact been superinfected through multiple sexual acts with the same partner. However, the frequency of transmission in the ZEHRP cohort (7 per 100 person years) is 1 in 300 to 1 in 600 unprotected coital acts [11 and unpublished], and so it would be unlikely that two independent transmissions would occur within the last seronegative to first seropositive time frame.

The observation, that multiple variants established infection at a higher frequency following epidemiologically unlinked transmission, suggested that factors capable of mitigating the severity of the genetic bottleneck could be more common in these cases. Examination of the clinical history of the newly infected partner in unlinked pairs revealed a striking association between the presence of multiple variants and vaginal/urethral discharge or lower abdominal pain (Table 4). As the numbers of transmission events for the unlinked recipients is small, this analysis was extended to include all newly infected individuals examined from the Rwanda and Zambia cohorts to date. While the presence of multiple variants in the newly infected partner no longer correlated with the presence of vaginal or urethral discharge, it was significantly correlated with lower abdominal pain and the presence of genital inflammation or ulceration (Table 4). Furthermore, in these cohorts, gender does not predispose an individual to being infected by more than one variant (Table 3). The association between genital tract infections and multiple variants is consistent with a previous study in a subtype A infected Kenyan female sex worker cohort [23]. These investigators established significant correlations between the presence of sexually transmitted diseases (STDs) and multiple infecting variants, but they also observed that over 90% of individuals with multiple variants had signs of a genital tract infection. This observation may account for the high frequency of heterogeneous virus present in newly infected individuals in sex-worker cohorts [5],[8], or those identified in a STD clinic [6], since both of these groups are likely to display a higher frequency of STDs than monogamous transmission pairs.

Genital tract inflammation could compromise the mucosal barrier during sexual exposure such that multiple variants can be transmitted and ultimately establish a systemic infection. While HIV-1 transmission from a chronically infected individual to their partner has been documented to be a relatively rare event (1 in 300–1000 unprotected sexual acts, [11],[13],[24],[25]), multiple studies have suggested that the presence of STDs in the at-risk individual increases susceptibility to HIV-1 infection [reviewed in 26],[27]. Genital tract infections could produce lesions in the mucosal surface that would increase access of virus to underlying target cells, thereby increasing the probability that multiple variants would gain access to distant sites and establish a systemic infection. Additionally, genital inflammation could provide an influx of activated potential target cells to the genital mucosa. It should be noted, however, that in a majority of HIV-1 transmission events, infection by a single variant occurs in the absence of diagnosed genital disease, arguing that HIV-1 can successfully cross the mucosal surface in the absence of such mucosal insults. This is consistent with previous epidemiological studies in the ZEHRP cohort [13].

### Nature of the Severe Genetic Bottleneck

Studies in rhesus macaques in which relatively large doses of a virus quasispecies have been applied to hormonally thinned vaginal mucosa showed that multiple viruses initiate a localized infection, but that only a subset is able to establish a systemic infection [28],[29]. These studies suggest that multiple variants may be able to initiate foci of infection within the genital compartment, but only a fraction of these can extend the infection beyond the mucosa. In the current study, we cannot differentiate between an “outgrowth” model in which multiple variants initially establish a localized infection and only a single variant can generate a systemic infection, and a “mucosal barrier” model in which the genetic bottleneck is the result of a single HIV-1 variant with the capacity to penetrate the mucosal barrier. The observed effect of genital inflammation or ulceration on transmission of multiple variants could result from an increase in the overall frequency of localized infections or a more permeable portal of entry. Nevertheless, it is clear that a single variant does not universally emerge as the most-fit virus, making it unlikely that the genetic bottleneck observed in the majority of linked transmission events is simply a test of viral replication fitness, as has been previously proposed [30].

That a single variant from the donor quasispecies, or a genetically restricted subset of variants, establishes infection in the cohorts studied here has direct implications for interventions aimed at preventing transmission of HIV-1. Irrespective of the basis for the genetic bottleneck, the earliest stage of HIV-1 infection represents a narrow window of time when there is highly restricted diversity in the virus population in which to implement protective strategies. Additional studies aimed at understanding the traits that confer the capacity to transmit and establish infection will be critical for guiding these preventative strategies.

## Materials and Methods

### Study Subjects

The transmission pairs characterized in this study were enrolled in the Zambia Emory HIV research Project (ZEHRP) in Lusaka or Projet San Francisco (PSF) Kigali, which provide voluntary HIV-1 testing and counseling, long-term monitoring and health care to cohabitating heterosexual couples in the capital cities of Zambia and Rwanda [10],[11]. At each visit the seronegative partner is tested for seroconversion using Determine and Unigold rapid HIV antibody tests [13]. Banked plasma samples from the antibody negative partners were tested for the presence of p24 antigen using the Beckman Coulter HIV-1 p24 antigen ELISA to identify individuals during acute infection [14],[15],[31]. The p24-positive subjects were tested within a 2 week window for seroconversion. Transmission pairs were prioritized from all seroconversion events by selecting first those who were identified as p24-positive and then those in which the recipients had a seronegative test date within the previous 3 or 4 months. Within these selection criteria, we balanced the number of FTM and MTF transmissions in order to avoid any gender bias. There were no transmission pairs for which we were unable to amplify the *env* region in both partners. Details regarding time since enrollment in the cohort, age and viral load at the time of transmission (where available) for the donor partners are provided in Table S1, Protocol S1. For the unlinked transmission recipients, which comprise only 13% of transmissions, we used similar selection criteria. Viral RNA was extracted from plasma and genomic DNA was extracted from uncultured peripheral blood mononuclear cells (PBMC) using blood samples collected by venipuncture into acid citrate dextrose tubes from both partners on the day at which infection was confirmed, as described previously [9]. Plasma samples that were p24 positive were analyzed when available. Informed consent and human subjects protocols were approved by the Emory University Institutional Review Board, the University of Zambia School of Medicine Research Ethics Committee and the Rwanda Ethics Committee.

The estimated number of days between infection and sample collection for each newly infected individual was calculated according the following algorithm. For individuals who were identified as antibody positive and p24 antigen negative at the date of sample collection, the estimated date of infection is presented as less than the number of days between the last seronegative test date and the first seropositive test date (day of sample collection). For individuals identified as antibody positive and p24 antigen positive at the date of seroconversion, the estimated date of infection was determined as 31 days prior to the date of seropositive testing (Fiebig stages III and IV, [18]). For individuals identified as antibody negative and p24 antigen positive, the estimated date of infection was determined as 22 days prior to that date (Fiebig stage II), and finally for individuals identified as viral RNA positive and antibody/p24 antigen negative, the estimated date of infection was determined as 17 days prior to this sample date (Fiebig stage I).

### End-Point Dilution, Single Genome Amplification of HIV-1 *env* Genes

For amplification of proviral HIV-1 *env* genes, genomic DNA was extracted from uncultured peripheral blood mononuclear cells (PBMC) using the Qiagen DNA Blood Mini Kit. For amplification of plasma HIV-1 *env* genes, RNA was purified from plasma samples using the Qiagen Viral Isolation Kit and cDNA prepared using the SuperScript III reverse transcriptase according to the manufacturer's instructions. All reverse transcription reactions were primed using an HIV-specific primer (OFM19, see sequence below). We were able to amplify viral sequences for all individuals from whom we attempted PCR amplification.

Full-length gp160 amplicons were obtained from genomic DNA or plasma cDNA using nested PCR amplification by single genome amplification [32]. Input genomic DNA or plasma cDNA was diluted to a limiting concentration such that approximately one-third or less of all second-round reactions produced a positive *env* amplicon. At this dilution, approximately 90% of the reactions will have originated from a single virus genome in the reaction [33]. Nested PCR amplifications were performed using Expand High Fidelity polymerase according to the manufacturer's instructions. Full-length gp160 amplification was performed using the following primer combinations: First round: sense primer Vif1 (5′ – GGGTTTATTACAGGGACAGCAGAG – 3′), antisense primer OFM19 (5′ – GCACTCAAGGCAAGCTTTATTGAGGCTTA – 3′); second round: sense primer EA1 (5′ – CCTAGGCATTTCCTATGGCAGGAAGAAGC – 3′), antisense primer EN1 (5′ – TTGCCAATCAGGGAAGTAGCCTTGTGT – 3′). PCR conditions were performed in 50 µl reaction volume with cycling parameters as previously published [9].

A portion of the final PCR product was analyzed by 1% agarose gel electrophoresis. Positive PCR reactions were purified using the QIAquick PCR Product Purification Kit or Exosap according to the manufacturer's instructions. Amplicons were directly sequenced by Lone Star Labs, Inc. (Houston, TX), Macrogen, Inc. (Seoul, Korea) or the UAB CFAR DNA Sequence Analysis Core (Birmingham, AL). Sequencing primers: For14 (5′–TATGGGACCAAAGCCTAAAGCCATGTG–3′) and Rev16 (5′–ATGGGAGGGGCATACATTGCT–3′) were used to generate overlapping sequences of the V1–V4 region of the HIV envelope.

### Sequence Analysis

To confirm PCR amplification of a single template, nucleotide sequence chromatograms were examined for multiple peaks, and any such amplicons were discarded. Sequences were trimmed to the first cysteine in V1 and the final cysteine in V4 for further analysis. Sequences were hand-aligned using MacClade 4.06 then gap-stripped to generate neighbor-joining trees using Clustal W. Unrelated reference sequences from the Los Alamos National Laboratory HIV Sequence Database were used as an outgroup for phylogenetic trees. Reference sequences represented Subtype A sequences from Rwanda and Subtype C sequences from Zambia (Subtype A: AY669706, AY669702, U86544, AB287377, AY713406, AB287376 and Subtype C: AB254143, AB254146, AY805330, AF286224, AF286225). The reliability of branching orders was assessed by bootstrap analysis with 1,000 replicates. Nucleotide and amino acid pairwise distance calculations for each recipient virus population was determined using PAUP 4.0b10. The Highlighter tool (Los Alamos National Laboratory HIV Sequence Database, http://www.hiv.lanl.gov/content/hiv-db/HIGHLIGHT/highlighter.html) was used to further analyze the diversity in viral variants transmitted to the recipient partner. Aligned sequences for each recipient were imported into the Highlighter tool and the master was selected as the sequence representing the most highly abundant variant in that virus population. Most recent common ancestor (MRCA) calculations were determined as previously described [21]. All V1–V4 *env* sequences from each newly infected and chronically infected individual were deposited in GenBank under the accession numbers FJ185853-FJ187678. Full length gp160 sequences have been deposited for ZM184F (EU166413-EU166438), ZM215F (EU166544-EU166575), ZM229M (EU166576-EU166604), ZM247F (EU166779-EU166856), and ZM249M (EU166857-EU166916) [20].

### Analysis of Recipient Clinical Histories

Medical and laboratory signs and symptoms of sexually transmitted infections (STI) were recorded systematically at routine quarterly study visits and at interim sick visits, with full physical and/or genital exams conducted as clinically indicated; physical and genital exams were routinely conducted on the visit date when lab test results indicated HIV-1 seroconversion. A self-reported symptom was considered present whether or not the patient sought medical treatment and included treatment administered at external clinics. Composite variables were created from physical exam, medical and laboratory data for genital ulceration and genital inflammation. An STI sign or symptom was included in this analysis if it was reported on the visit date when HIV-1 seroconversion was detected or within one quarterly study visit prior to this visit date. Base SAS® and SAS/STAT® statistical software was used for data management and analysis; Fisher's exact test was used to assess statistical significance of difference in STI proportions between groups.

## Supporting Information

Figure S1 Diversity in individuals infected with multiple variants. Aligned linked recipient sequences were analyzed by the Highlighter tool (Los Alamos National Laboratory website - HIV Sequence Database) for (A) RW57F and (B) ZM229F (C) ZM224F and (D) ZM215F. Tic marks indicate nucleotide differences from the indicated master sequences derived from the recipient. Nucleotide differences are color-coded and are marked according to their genetic location along the length of V1–V4. Colors are as follows: A: green, T: red, G: yellow, C: blue and gaps: gray. Red box indicates signature of 5 synonymous nucleotide differences between variants that established infection in RW57F.
(1.72 MB PDF)

Figure S2 Diversity in newly infected individuals. Aligned linked recipient sequences were analyzed by the Highlighter tool (Los Alamos National Laboratory website - HIV Sequence Database) for (A) ZM216M and (B) ZM292M. Tic marks indicate nucleotide differences from the indicated master sequences derived from the recipient. Nucleotide differences are color-coded and are marked according to their genetic location along the length of V1–V4. Colors are as follows: A: green, T: red, G: yellow, C: blue and gaps: gray. Red box indicates CTL-escape footprint.
(1.36 MB PDF)

Table S1 Enrolled Partner
(0.05 MB PDF)

Protocol S1 Criteria for defining infection by a single or multiple variant
(0.04 MB PDF)
