# Supplementary figures and images for: Inflammatory Genital Infections Mitigate a Severe Genetic Bottleneck in Heterosexual Transmission of Subtype A and C HIV-1
Source: PLoS Pathog. 2009 Jan 23;5(1):e1000274. doi: 10.1371/journal.ppat.1000274 (PMC2621345; doi:10.1371/journal.ppat.1000274)

**A**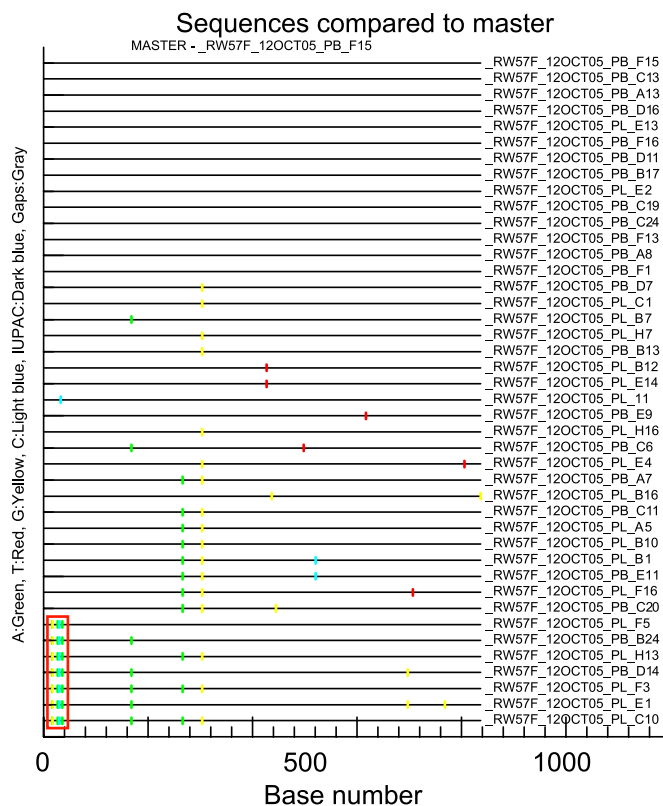**B**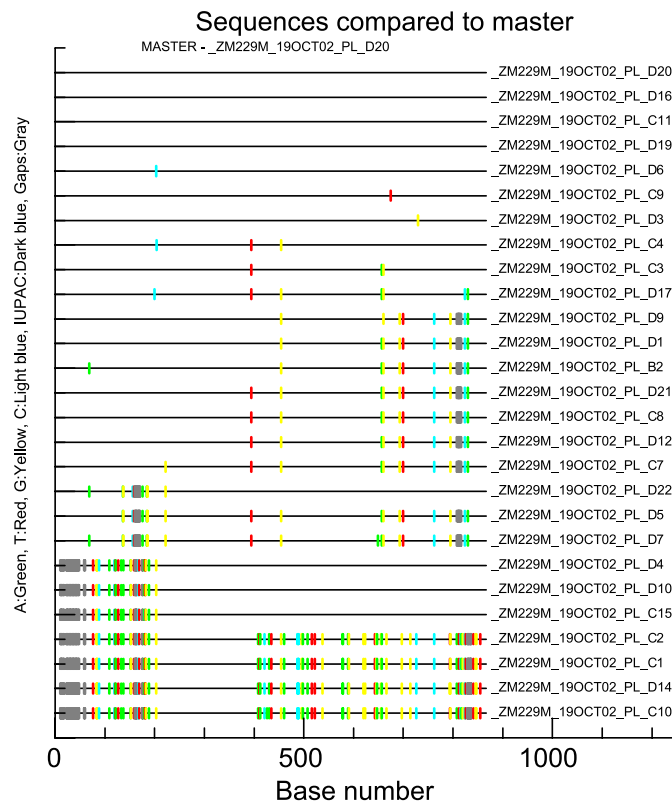**C**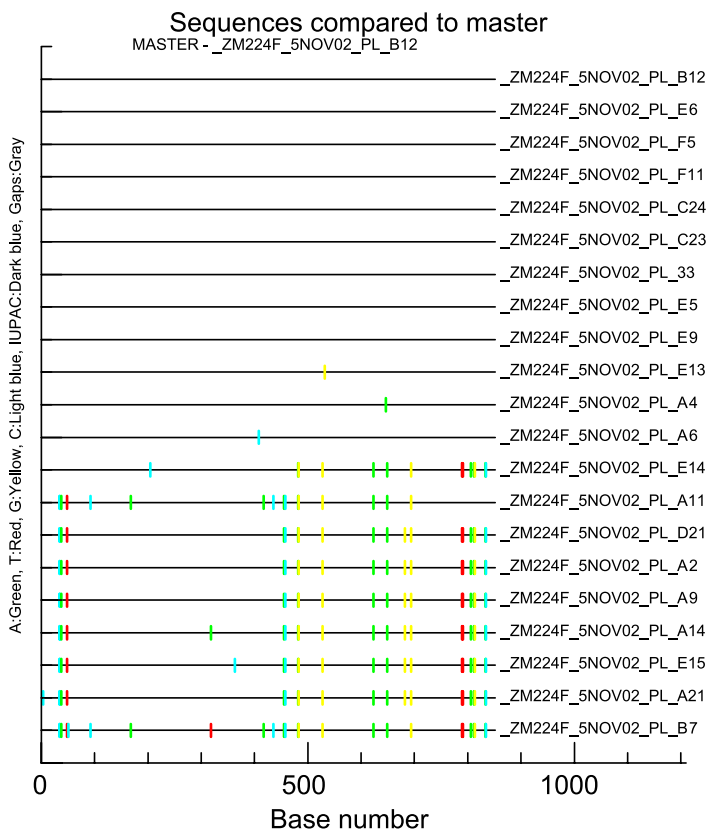**D**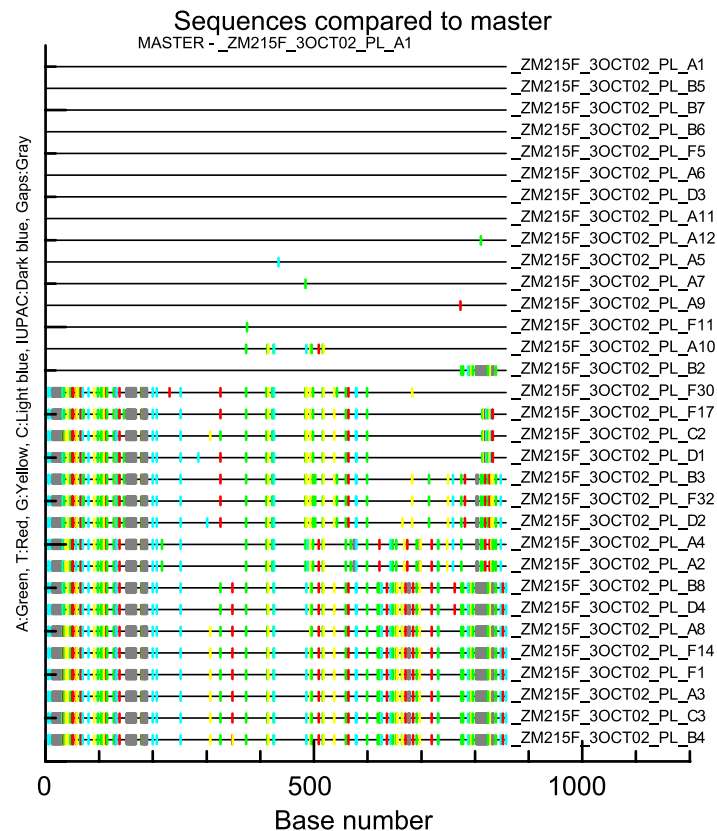

Supplement: Figure S1 — Diversity in individuals infected with multiple variants. Aligned linked recipient sequences were analyzed by the Highlighter tool (Los Alamos National Laboratory website - HIV Sequence Database) for (A) RW57F and (B) ZM229F (C) ZM224F and (D) ZM215F. Tic marks indicate nucleotide differences from the indicated master sequences derived from the recipient. Nucleotide differences are color-coded and are marked according to their genetic location along the length of V1–V4. Colors are as follows: A: green, T: red, G: yellow, C: blue and gaps: gray. Red box indicates signature of 5 synonymous nucleotide differences between variants that established infection in RW57F. (1.72 MB PDF) [file ppat.1000274.s001.pdf]

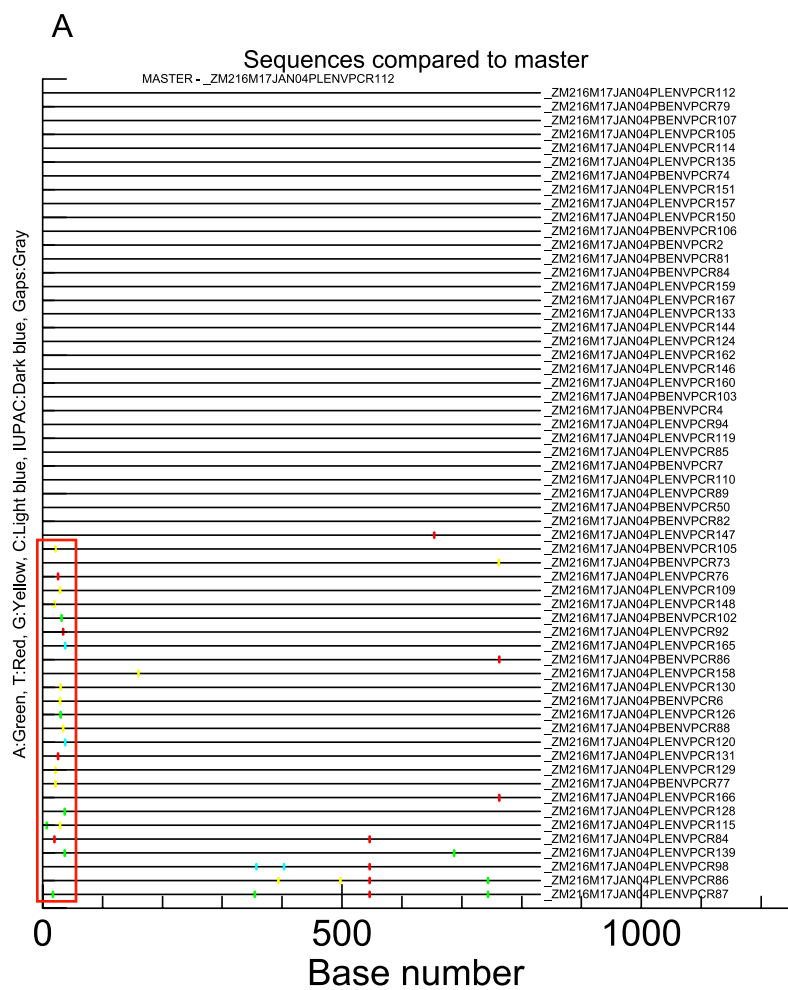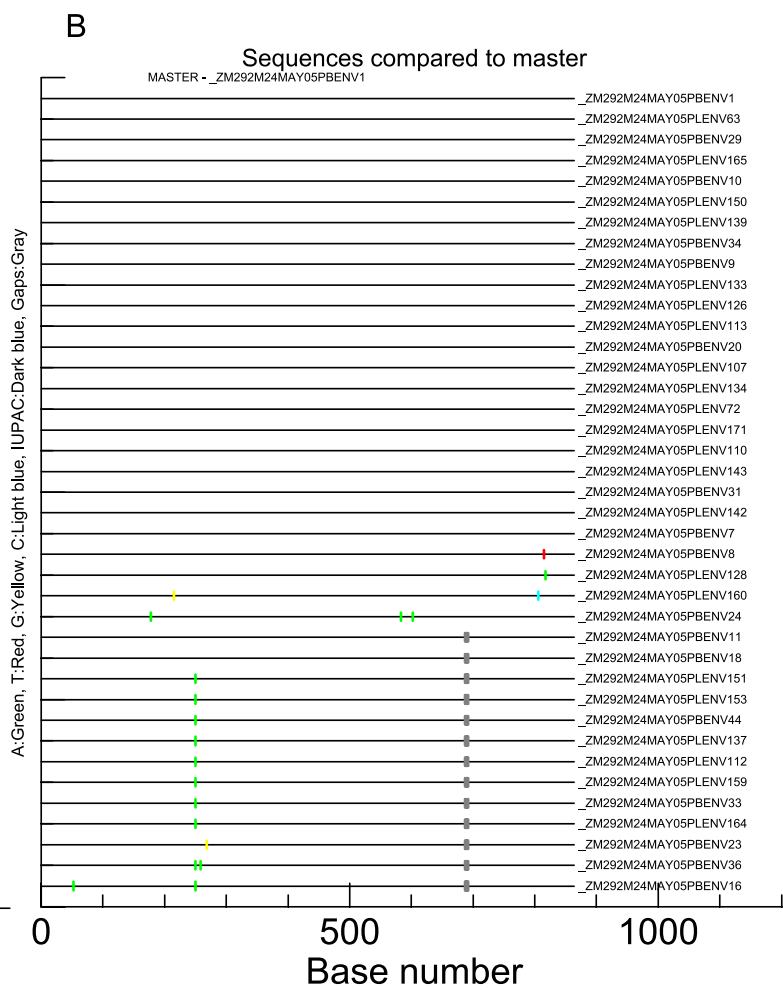

Supplement: Figure S2 — Diversity in newly infected individuals. Aligned linked recipient sequences were analyzed by the Highlighter tool (Los Alamos National Laboratory website - HIV Sequence Database) for (A) ZM216M and (B) ZM292M. Tic marks indicate nucleotide differences from the indicated master sequences derived from the recipient. Nucleotide differences are color-coded and are marked according to their genetic location along the length of V1–V4. Colors are as follows: A: green, T: red, G: yellow, C: blue and gaps: gray. Red box indicates CTL-escape footprint. (1.36 MB PDF) [file ppat.1000274.s002.pdf]
