# Supplementary material for: Inflammatory Genital Infections Mitigate a Severe Genetic Bottleneck in Heterosexual Transmission of Subtype A and C HIV-1
Source: PLoS Pathog. 2009 Jan 23;5(1):e1000274. doi: 10.1371/journal.ppat.1000274 (PMC2621345; doi:10.1371/journal.ppat.1000274)
Supplement: Table S1 — Enrolled Partner (0.05 MB PDF) [file ppat.1000274.s003.pdf]

Table S1 - Enrolled Partner

Subject	Age (years) at Seroconversion	Time (months) enrolled	MKC stage[a]	Viral Load
ZM190M	29.2	75	1	248986
ZM198M	34.9	45	1	31362
ZM201F	25.5	107	1	471382
ZM205M	30.6	18	1	47133
ZM216F	32.8	21	1	371901
ZM221F	30.5	6	1	62741
ZM229F	22.3	3	1	50000
ZM238F	45.0	1	4	95545
ZM242M	26.3	3	4	2974
ZM243M	33.3	15	1	302612
ZM248M	25.4	3	3	10863
ZM292F	21.3	27	1	7165
RW19M	30.0	18	1	NA
RW35F	23.0	12	1	NA
RW36F	23.0	12	4	NA
RW41F	40.5	6	1	NA
RW53M	41.5	18	1	NA
RW56M	26.1	12	1	NA
RW57M	29.5	42	3	NA
RW67F	29.5	12	1	26515

[a]Modified Kigali Combined staging system (Lifson, A. R., S. Allen, W. Wolf, A. Serufilira, G. Kantarama, C. P. Lindan, E. S. Hudes, F. Nsengumuremyi, H. Taelman, and J. Batungwanayo. 1995. Classification of HIV infection and disease in women from Rwanda. Evaluation of the World Health Organization HIV staging system and recommended modifications. Ann Intern Med 122:262-70; Peters, P. J., I. Zulu, N. G. Kancheya, S. Lakhi, E. Chomba, C. Vwalika, D. J. Kim, I. Brill, J. Meinzen-Derr, A. Tichacek, and S. A. Allen. 2008. Modified Kigali combined staging predicts risk of mortality in HIV-infected adults in Lusaka, Zambia. AIDS Res Hum Retroviruses 24:919-24.) MKC stage is similar to the clinical measures in WHO staging.
