# Supplementary material for: Inflammatory Genital Infections Mitigate a Severe Genetic Bottleneck in Heterosexual Transmission of Subtype A and C HIV-1
Source: PLoS Pathog. 2009 Jan 23;5(1):e1000274. doi: 10.1371/journal.ppat.1000274 (PMC2621345; doi:10.1371/journal.ppat.1000274)
Supplement: Protocol S1 — Criteria for defining infection by a single or multiple variant (0.04 MB PDF) [file ppat.1000274.s004.pdf]

Protocol S1:

Criteria for defining infection by a single or multiple variant:

When an individual is infected with a single genetic variant the diversification observed is the result of random RT-initiated mutations that are scattered across the *env* sequence. The model used to calculate the most recent common ancestor is based on this observation and yields a Poisson distribution of mutations with star-like phylogeny [21]. In contrast, in each of the individuals that have been classified as being infected with multiple variants, there are at least 2 subsets of sequences in which the members exhibit multiple identical nucleotide differences. This yields a non-random distribution of sequences that results in MRCAs that significantly exceed the maximum estimated date of infection. An example of this is shown in Figure 4B for Z247F, where there are two distinct homogeneous sequence populations that differ from each other in more than 20 positions. Similar results were observed for each of the individuals classified as being infected by multiple variants and the highlighter output for each of these is presented in Supplementary Figure 1. Even in the case of RW57, where two very similar viruses initiated infection, the two variant populations differ by a cluster of five synonymous mutations in the V1 region of gp120 that differentiate the seven sequences of cluster 2 from the 35 in cluster 1. Moreover, these signature sequences are shared by two of the donor *env* genes, indicating that they did not arise de novo in the recipient.

Thus based on a combination of MRCA calculations, distinct populations of sequences in the highlighter analysis, and in the case of transmission pairs more than one branch from the donor tree, it was possible to unambiguously define individuals as being infected by more than one donor variant even when the donor sequences were not available.
